# Ivermectin Treatment for Cattle Reduced the Survival of Two Malaria Vectors, *Anopheles dirus* and *Anopheles epiroticus*, Under Laboratory Conditions in Central Vietnam

**DOI:** 10.4269/ajtmh.20-1239

**Published:** 2021-04-26

**Authors:** Estee Y. Cramer, Nguyen Xuan Quang, Jeffrey C. Hertz, Do Van Nguyen, Huynh Hong Quang, Ian Mendenhall, Andrew A. Lover

**Affiliations:** 1 Department of Biostatistics and Epidemiology, School of Public Health and Health Sciences, University of Massachusetts-Amherst, Amherst, Massachusetts;; 2 Institute for Malariology, Parasitology and Entomology, Ministry of Health, Quy Nhon, Vietnam;; 3 U.S. Naval Medical Research Unit TWO, Singapore;; 4 Programme in Emerging Infectious Diseases, Duke-NUS Medical School, Singapore

## Abstract

Ivermectin is a low-cost and nontoxic mosquitocide that may have a role in malaria elimination. However, the extent to which this drug impacts the mortality of *Anopheles dirus* and *Anopheles epiroticus*, two important malaria vectors in Southeast Asia, is unknown. This study compared and quantified anopheline mortality after feeding on ivermectin-treated cattle and control cattle in Vietnam. Local anopheline colonies fed on cattle 1 to 3, 6 to 8, 13 to 15, 20 to 22, and 28 to 30 days after injection (DAI) with ivermectin (intervention) or saline (control). *An. dirus* that fed on ivermectin-treated cattle had higher mortality rates than controls for up to 20 DAI (*P* < 0.05); *An. epiroticus* that fed on ivermectin-treated cattle had consistently higher mortality rates than controls for up to 8 DAI (*P* < 0.05). Feeding on ivermectin-treated cattle increased the mortality rate of these vector species for biologically relevant time periods. Therefore, ivermectin has the potential to become an important tool for integrated vector management.

Throughout the Greater Mekong Subregion (GMS), malaria transmission occurs primarily in forested areas and along international borders. In these areas, members of the Dirus and Sundaicus complexes (including *Anopheles epiroticus* and *An. dirus*) are important vectors for malaria transmission.^1^ Although great progress has been made with widescale use of long-lasting insecticide-treated bed nets, artemisinin-based combination therapies, and rapid diagnostic tests, these are not sufficient to eliminate malaria in some areas. Changes in mosquito behavior and decreased sensitivity to insecticides have hindered malaria elimination efforts in the GMS, necessitating new tools to address complex vector bionomics.^2^ Ivermectin is a widely used general antiparasitic drug that has emerged as a novel intervention to target residual malaria transmission.^3^ Ivermectin is low-cost, has low toxicity at recommended doses in humans and cattle, and has a well-established regulatory environment. However, ivermectin has a short half-life in mammals.^4^ To explore use-case scenarios for programmatic use, data are needed to determine the duration of mosquitocidal activity in target vector species. Studies have been undertaken under semi-field conditions in Tanzania^5^ and under laboratory conditions in Southeast Asia^6^; however, data regarding Southeast Asia anopheline species are necessary to progress the research agenda. To address this gap, this study aimed to compare and quantify anopheline mortality after feeding on ivermectin-treated cattle and control cattle. This was performed in a laboratory experiment with field treatments in Central Vietnam using local colonies of *An. epiroticus* and *An. dirus*.

Animal use was approved by the National University of Singapore (IACUC #B18-0303) and by the University of Massachusetts (#IACUC 2019-0011). For each species-specific study, four local cattle (approximately 250 kg) were purchased after a health evaluation by local animal health staff. All eight animals were male beef cattle of the common Bo Vang (Vietnamese Yellow) zebu breed.^7^ Throughout the study, cattle were housed in outdoor pens, fed on grass, and were provided water *ad librium*; regular welfare checks were performed by animal health staff. Cattle were treated with albendazole for 2 weeks before ivermectin dosing to clear residual parasites and then randomized to a treatment group. Intervention cattle were administered a subcutaneous injection of 0.2 mg/kg (weight estimated from girth measurements) veterinary-grade “Vimectin” (Vemedim Corporation; Can Tho, Vietnam). Control cattle were administered a sham injection of saline. Simulation-based methods were used to determine the study sample size; both studies were powered at 0.9 to detect a relative survival difference (hazard ratio [HR]) of 50% at 21 days after injection (DAI). A minimum of 33 blood-fed vectors were required per animal per timepoint per trial, with an excess to compensate for any losses (partially engorged or potential mortality during blood-feeding).

The feeding process was conducted with 3- to 5-day old female mosquitoes reared from the Institute of Malariology Parasitology and Entomology Quy Nhon (IMPE-QN) colonies of *An. dirus* and *An. epiroticus*. Mosquitoes were maintained with a Hemotek membrane feeding system (Hemotek Ltd., Blackburn, United Kingdom) with porcine blood, and were sugar starved for 12 to 16 hours prior to feeding on cattle. For blood-feeding, mosquitoes were placed into six small cardboard containers with mesh-covered tops (approximately 20 per cup; total of 60 [minimum 50] per timepoint per animal). Cups were placed on the flank of the cow and held for approximately 30 minutes. After this period, mosquitoes were evaluated for repletion. Fully engorged mosquitoes were removed with an aspirator and transferred to maintenance cups with a 10% sucrose solution where they were monitored daily for mortality. The feeding process was repeated at 1 to 3, 6 to 8, 13 to 15, 20 to 22, and 28 to 30 DAI with ivermectin (intervention) or saline (control). This entire process was repeated for the second vector species experiments. After data collection, cattle were pastured for 35 days to ensure clearance of all traces of ivermectin before being resold to owners.^8^ A schematic of the study design is shown in Figure 1. Data collection was conducted from March 2019 to March 2020.

**Figure 1.**
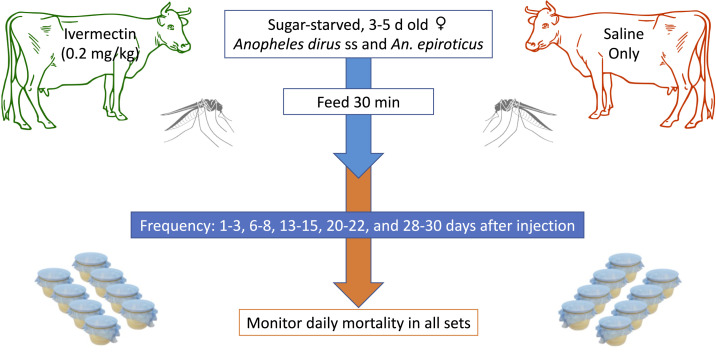
Overview of study design. This figure appears in color at www.ajtmh.org.

Flexible parametric models were used to quantify the survival of mosquitos at each DAI. To estimate HRs, individual models were adjusted for treatment group (intervention compared to control) and for trial number (trial 1 compared to trial 2). The optimal number of splines per model was determined using the Akaike Information Criteria^9^; individual models included between zero and four splines. Additionally, the difference in median survival time was calculated for the intervention and control mosquitos for each DAI. All analyses were conducted in R 3.5.1.^10^

Trials for *An. dirus* monitored a total of 2116 control mosquitos and 2173 intervention mosquitos for mortality. Full mortality was observed for all replicates, with the exception of 12 of 60 replicates for *An. dirus*, which were administratively censored 6 to 14 days after feeding. Kaplan-Meier curves comparing the survival probability for mosquitos are shown (Figure 2A). For *An. dirus*, there was a significant difference (*P* < 0.05) in the survival for each treatment group through 20 DAI. There was no evidence of differences in survival at timepoints after this, with the exception of 22 DAI. At 1 DAI (adjusted hazard ratio [aHR], 3.08; 95% confidence interval [CI], 2.34–4.05) and 2 DAI (aHR, 3.49; 95% CI, 2.62–4.62), the survival probability ratios were greatest (Figure 3A). At later DAI, the HRs of intervention to control survival probability tended toward the null. The median difference in survival time was also greatest at 1 DAI (median difference of 7 days; 95% CI, 4–8 days) and at 2 DAI (median difference of 5 days; 95% CI, 4–7 days) (Supplemental Table 3).

**Figure 2.**
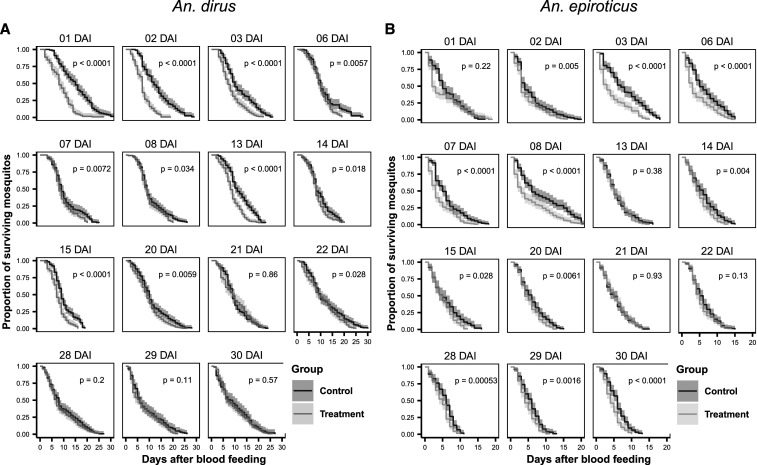
Kaplan-Meier survival plots for vector, *Anopheles dirus* (**A**) and *An. epiroticus* (**B**), survival in ivermectin-treated and control cattle by feeding day. Kaplan-Meier plots of anopheline survival when fed on treated and control cattle at different days after injection of 0.2 mg/kg of ivermectin. Black lines represent the survival of mosquitos fed on the control cattle. Gray lines represent the mosquitos that fed on the cattle treated with ivermectin. The survival differences were compared using a log-rank test.

**Figure 3.**
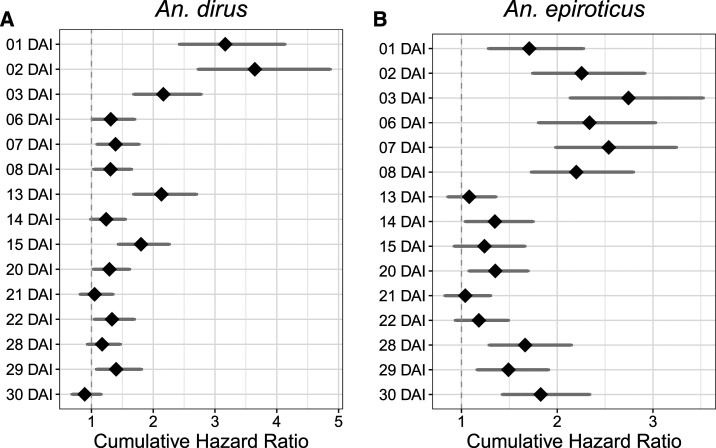
Quantitative comparison of hazard ratios (HRs) for vector, *Anopheles dirus* (**A**) and *An. epiroticus* (**B**), survival in ivermectin-treated and control cattle by feeding day. Cumulative HRs of anopheline survival when fed on treated and control cattle at different days after injection of 0.2 mg/kg of ivermectin are shown. Black diamonds represent the cumulative HR. Dark gray lines represent the 95% confidence interval.

Trials for *An. epiroticus* monitored 2003 control mosquitos and 2144 intervention mosquitos. Kaplan-Meier curves were used to compare the survival probability at each DAI (Figure 2B). There was a significant difference (*P* < 0.05) in the survival probability ratio of the intervention group compared with the control group on all DAI with the exceptions of 13, 15, 21, and 22 DAI. For *An. epiroticus*, the survival probability difference was greatest from 2 DAI to 8 DAI. During this timeframe, the HRs were consistently greater than 2.0 (Figure 3B). The maximum difference in survival probability was observed at 3 DAI feeding groups (aHR, 2.74; 95% CI, 2.14–3.52). The largest difference in median survival was also observed at 3 DAI (median difference of 4 days; 95% CI, 2–6 days) (Supplemental Table 4). In contrast to the *An. dirus* trials, no linear relationship was evident between the DAI and the effect size of the HRs of the intervention and control groups. After 8 DAI, the magnitude of the HRs diminished and remained close to null through 22 DAI. However, a second peak of “late mortality” was observed at 28 to 30 DAI. Although there was a statistically significant difference in survival probabilities, the biological mechanism for this observed effect is uncertain.

To assess whether ivermectin may cause mosquito repellency, a groupwise post hoc χ^2^ test of association with a Bonferroni correction was used. For *An. dirus,* the magnitude and direction of significant differences were inconsistent between the treatment and control groups (Supplemental Table 5). For *An. epiroticus*, there was no significant difference in the proportion of mosquitos that fed in the treatment and control groups at any DAI (Supplemental Table 6). These post hoc results do not support any difference in feeding rates with ivermectin in these studies.

This study was not without limitations. These experiments used forced feeding; therefore, vectors fed only at a single time point and only fully engorged mosquitos were analyzed. This could have led to overestimates of the survival difference between the intervention and control groups. Additionally, we did not evaluate differences in vector fecundity after ivermectin treatment, which has the potential for longer-lasting impacts on the overall vector populations.

This study demonstrated, for the first time in Southeast Asia, that treating cattle with ivermectin at standard veterinary dosages led to reduced survival of *An. epiroticus* and *An. dirus*, which are two important malaria vectors in the GMS. Decreased mortality was observed for moderate time periods (20 DAI for *An. dirus* and 8 DAI for *An. epiroticus*), presumably because of the limited half-life of ivermectin. Data regarding membrane feedings with *An. dirus* suggested plausible blood levels in humans are likely too low to impact the mortality of feeding anophelines after approximately 1 day.^6^ Our results support previous suggestions that cattle may provide a viable delivery mode for ivermectin in specific settings. Furthermore, these results are broadly consistent with those of cattle feeding studies performed in Tanzania using the unrelated vector *An. arabiensis*.^5^ However, ivermectin decreased survival for nearly 28 days for *An. arabiensis*.^5,11^

Major progress has been made toward malaria elimination in the GMS, but novel methods are urgently needed to address areas with hypo-endemic and meso-endemic transmission, especially in areas where vector feeding times have shifted because of reduced host availability via insecticide-treated bednets.^12^ Although *An. epiroticus* is generally more zoophilic than *An. dirus*, anthropophilic populations of both species exist and are important malaria vectors.^13–15^ Moreover, cattle ownership was found to be independently associated with approximately 2.5-fold greater exposure to *Anopheles* spp. in some settings in the GMS,^16^ thus providing a clear path to targeting residual malaria transmission in populations at highest risk.

Limited data suggest that the GMS may be an ideal setting for zooprophylaxis-aided ivermectin-based vector elimination. Although the mortality effects seen for *An. dirus* and *An. epiroticus* are shorter than those found for *An. arabiensis*,^5^ membrane feedings suggest high ivermectin susceptibility of *An. dirus.* Data are not directly comparable because of variations in feeding sources, however, the 10-day LC_50_ for *An. dirus* is lower than that of *An. arabiensis* (3.7 ng/ml).^17^ Additionally, the high density of the Viet cattle breed in areas with residual transmission makes the GMS an excellent candidate for zooprophylaxis-aided ivermectin-based vector elimination (ZAIVE). Recent cross-comparisons suggest that zebu cattle have longer ivermectin residence times relative to other cattle because of greater body lipophilicity.^18^ Deploying ivermectin via zooprophylaxis-aided approaches is a promising method to target residual malaria transmission. The broader antihelminth activities of ivermectin also contribute to animal health via a genuine One Health approach.

Future studies are needed to gain a fuller understanding of cattle breed-specific effects and to determine whether a sufficient density of ivermectin-treated cattle in peri-domestic settings can impact village-level anopheline vector populations. These and related field studies are underway in the Central Highlands of Vietnam.

## Supplemental figure and tables

Supplemental materials
