# Supplementary material for: Ivermectin Treatment for Cattle Reduced the Survival of Two Malaria Vectors, Anopheles dirus and Anopheles epiroticus, Under Laboratory Conditions in Central Vietnam
Source: Am J Trop Med Hyg. 2021 Apr 26;104(6):2165–8. doi: 10.4269/ajtmh.20-1239 (PMC8176477; doi:10.4269/ajtmh.20-1239)
Supplement: Supplementary file 1 [file tpmd201239.SD1.docx]

Supplemental Table 1. Cumulative hazard ratios, *Anopheles dirus*

**Notes**: Shown are the cumulative hazard ratioswith 95% confidence intervals for the intervention vs. control group at each day after injection with ivermectin based on flexible parametric models with 0–4 splines. Hazard ratios (HR) greater than 1 indicate a shorter survival time for the intervention group compared to the control group. The groups in which there was a significant difference in survival between the intervention and control group are bolded (*P*< 0.05). Data from this table corresponds to Figure 3, Panel A.

| Group | HR | 95% CI |
| --- | --- | --- |
| **DAI 01** | 3.17 | (2.43, 4.13) |
| **DAI 02** | 3.64 | (2.73, 4.86) |
| **DAI 03** | 2.16 | (1.69, 2.77) |
| **DAI 06** | 1.31 | (1.01, 1.70) |
| **DAI 07** | 1.39 | (1.09, 1.77) |
| **DAI 08** | 1.31 | (1.04, 1.65) |
| **DAI 13** | 2.13 | (1.69, 2.77) |
| DAI 14 | 1.24 | (0.99, 1.55) |
| **DAI 15** | 1.80 | (1.44, 2.26) |
| **DAI 20** | 1.29 | (1.03, 1.61) |
| DAI 21 | 1.05 | (0.82, 1.35) |
| **DAI 22** | 1.33 | (1.05, 1.69) |
| DAI 28 | 1.17 | (0.94, 1.47) |
| **DAI 29** | 1.40 | (1.08, 1.81) |
| DAI 30 | 0.89 | (0.69, 1.15) |

Supplemental Table 2: Cumulative hazard ratios, *Anopheles epiroticus*

**Notes**: Supplemental Table 2 shows the cumulative hazard ratios and 95% confidence intervals for the intervention compared to the control group at each day after injection with ivermectin based on flexible parametric models with zero to four splines. Hazard ratios greater than 1 indicate a shorter survival time for the intervention group compared to the control group. The groups in which there was a significant difference in survival between the intervention and control group are bolded (*P*< 0.05). Data from this table corresponds to Figure 3, Panel B.

| Group | HR | 95% CI |
| --- | --- | --- |
| **DAI 01** | 1.71 | (1.28, 2.27) |
| **DAI 02** | 2.25 | (1.75, 2.91) |
| **DAI 03** | 2.74 | (2.14, 3.52) |
| **DAI 06** | 2.34 | (1.81, 3.03) |
| **DAI 07** | 2.54 | (1.99, 3.24) |
| **DAI 08** | 2.20 | (1.73, 2.79) |
| DAI 13 | 1.08 | (0.86, 1.36) |
| **DAI 14** | 1.35 | (1.04, 1.75) |
| DAI 15 | 1.24 | (0.93, 1.66) |
| **DAI 20** | 1.35 | (1.08, 1.69) |
| DAI 21 | 1.04 | (0.83, 1.30) |
| DAI 22 | 1.18 | (0.94, 1.49) |
| **DAI 28** | 1.67 | (1.29, 2.15) |
| **DAI 29** | 1.49 | (1.17, 1.91) |
| **DAI 30** | 1.83 | (1.43, 2.35) |

Supplemental Table 3: Median survival times, *Anopheles dirus.*

**Notes**: Supplemental Table 3 shows the median survival difference between the control group and ivermectin-treated group. Negative numbers indicate that the survival in the intervention group was lower than the control group. The intervals were calculated using a bootstrap approach to detect the difference between the survival medians for the intervention and groups. The days after infection with ivermectin in which the 95% confidence intervals are significantly different between groups are bolded. For *An. dirus*, there is a statistically significant difference in survival for 01, 02, 03, 13, and 15 days after injection.

| Group | Control mosquitos (n) | Treated mosquitos (n) | Difference in median survival (days) | 95% CI |
| --- | --- | --- | --- | --- |
| 01 DAI | 132 | 123 | -7 | (-8,-4) |
| 02 DAI | 115 | 150 | -5 | (-7,-4) |
| 03 DAI | 140 | 144 | -3 | (-4,-1) |
| 06 DAI | 119 | 144 | 0 | (-1,1) |
| 07 DAI | 134 | 146 | -1 | (-2,0) |
| 08 DAI | 160 | 151 | 0 | (-1,1) |
| 13 DAI | 140 | 175 | -2 | (-5,-1) |
| 14 DAI | 145 | 178 | 0 | (-2,0) |
| 15 DAI | 182 | 148 | -2 | (-3,-1) |
| 20 DAI | 155 | 166 | -1 | (-2,0) |
| 21 DAI | 152 | 112 | 0 | (-2,2) |
| 22 DAI | 154 | 130 | 0 | (-2,3) |
| 28 DAI | 155 | 156 | -1 | (-2,0) |
| 29 DAI | 125 | 119 | 0 | (-3,2) |
| 30 DAI | 108 | 131 | -1 | (-4,3) |

Supplemental Table 4: Median survival times,*Anopheles epiroticus.*

**Notes**: Supplemental Table 4 shows the median survival difference between the control group and ivermectin-treated group. Negative numbers indicate that the survival in the intervention group was lower than the control group. The intervals were calculated using a bootstrapping approach to detect the difference between the survival medians for the intervention and control groups. The days after infection with ivermectin in which the 95% confidence intervals are significantly different between groups are bolded. For *An. epiroticus*, a significant difference in survival was observed at 01, 03, 06, 07, 08,and 30 days after injection.

| Group | Control mosquitos (n) | Treated mosquitos (n) | Difference in median survival (days) | 95% CI |
| --- | --- | --- | --- | --- |
| 01 DAI | 108 | 136 | -3 | (-4,-2) |
| 02 DAI | 135 | 158 | -1 | (-2,0) |
| 03 DAI | 134 | 160 | -4 | (-6,-2) |
| 06 DAI | 124 | 146 | -2 | (-4,-1) |
| 07 DAI | 144 | 153 | -2 | (-3,-2) |
| 08 DAI | 134 | 156 | -3 | (-6,-2) |
| 13 DAI | 156 | 146 | 0 | (-1,1) |
| 14 DAI | 117 | 130 | -1 | (-3,1) |
| 15 DAI | 101 | 103 | 0 | (-2,2) |
| 20 DAI | 160 | 158 | -1 | (-2,0) |
| 21 DAI | 165 | 140 | 0 | (-2,2) |
| 22 DAI | 144 | 146 | -1 | (-2,0) |
| 28 DAI | 121 | 132 | -1 | (-2,0) |
| 29 DAI | 130 | 135 | -2 | (-2,0) |
| 30 DAI | 130 | 145 | -2 | (-2,-1) |

Supplemental Figure 1: Median difference in survival between intervention and control groups stratified by day after injection with ivermectin for *An. dirus* and *An. epiroticus*

Supplemental figure 1 displays the median difference in survival between the intervention and control groups of mosquitos stratified by feeding day. Median values (black diamond) shown with 95% confidence intervals (gray error bars). Confidence intervals were calculated using a bootstrapping approach.

Supplemental Table 5: Comparison in proportion of bloodfed mosquitosbetween control and treatment groups, *An. dirus*.

**Notes**: Supplemental Table 5 shows the proportion of mosquitos that fed on the cattle.*P* values were calculated using a groupwise post hoc chi-squared test with a Bonferroni correction. There were statistically significant differences (adjusted *P*-value < 0.05) between the proportion of mosquitos that fed in the treatment group vs. the control group on 2, 13,14, 15, and 21 DAI, however the magnitude and direction of the effect varies.

| **Group** | **Proportion bloodfed, control group** | **Proportion bloodfed, treatment group** | ***P*-value (Bonferroni adjusted)** |
| --- | --- | --- | --- |
| 01 DAI | 0.55 | 0.51 | 1.00 |
| 02 DAI | 0.48 | 0.62 | 0.027 |
| 03 DAI | 0.58 | 0.60 | 1.00 |
| 06 DAI | 0.50 | 0.60 | 0.42 |
| 07 DAI | 0.56 | 0.61 | 1.00 |
| 08 DAI | 0.67 | 0.63 | 1.00 |
| 13 DAI | 0.58 | 0.73 | 0.016 |
| 14 DAI | 0.60 | 0.74 | 0.028 |
| 15 DAI | 0.76 | 0.62 | 0.017 |
| 20 DAI | 0.65 | 0.69 | 1.00 |
| 21 DAI | 0.63 | 0.47 | 0.0051 |
| 22 DAI | 0.64 | 0.54 | 0.49 |
| 28 DAI | 0.65 | 0.65 | 1.00 |
| 29 DAI | 0.52 | 0.50 | 1.00 |
| 30 DAI | 0.45 | 0.55 | 0.69 |

Supplemental Table 6: Comparison in proportion of bloodfed mosquitos between control and treatment groups,*An. epiroticus*

**Notes**: Supplemental Table 6 shows the proportion of mosquitos that fed on the cattle. *P*-values were calculated using a groupwise post hoc chi-squared test with a Bonferroni correction. There were no days with a statistically significant difference (adjusted *P*-value < 0.05) between the proportion of mosquitos that fed in the treatment group vs. the control group.

| **Group** | **Proportion bloodfed, control group** | **Proportion bloodfed, treatment group** | ***P*-value (Bonferroni adjusted)** |
| --- | --- | --- | --- |
| 01 DAI | 0.45 | 0.57 | 0.21 |
| 02 DAI | 0.56 | 0.66 | 0.59 |
| 03 DAI | 0.56 | 0.67 | 0.29 |
| 06 DAI | 0.52 | 0.61 | 0.80 |
| 07 DAI | 0.60 | 0.64 | 1.00 |
| 08 DAI | 0.56 | 0.65 | 0.75 |
| 13 DAI | 0.65 | 0.61 | 1.00 |
| 14 DAI | 0.49 | 0.54 | 1.00 |
| 15 DAI | 0.42 | 0.43 | 1.00 |
| 20 DAI | 0.67 | 0.66 | 1.00 |
| 21 DAI | 0.69 | 0.58 | 0.34 |
| 22 DAI | 0.60 | 0.61 | 1.00 |
| 28 DAI | 0.50 | 0.55 | 1.00 |
| 29 DAI | 0.54 | 0.56 | 1.00 |
| 30 DAI | 0.54 | 0.60 | 1.00 |
